# Examining aptitude and barriers to evidence-based medicine among trainees at an ACGME-I accredited program

**DOI:** 10.1186/s12909-020-02341-9

**Published:** 2020-11-10

**Authors:** Mai A. Mahmoud, Sa’ad Laws, Antoun Kamal, Dabia Al Mohanadi, Ahmed Al Mohammed, Ziyad R. Mahfoud

**Affiliations:** 1 Weill Cornell Medicine in Qatar, Education City, P.O. Box 24144, Doha, Qatar; 2 Hamad Medical Corporation, Doha, Qatar

**Keywords:** EBM practice, Trainees’ knowledge and attitude about EBM

## Abstract

**Background:**

The aims of Evidence-Based medicine (EBM) are to promote critical thinking and produces better patients’ outcome (Profetto-McGrath J, J Prof Nurs Off J Am Assoc Coll Nurs 21:364-371, 2005). Accreditation Council for Graduate Medical Education (ACGME) competencies require trainees to locate, appraise and apply clinical evidence to patients’ care. Despite the emphasis that ACGME place on EBM, few organizations provide adequate training in EBM. This is even more critical in regions where medical trainees matriculate from diverse backgrounds of undergraduate medical education, where EBM may not be emphasized nor taught at all. EBM practice has a history of research in the West, however, EBM has not been widely studied in the Middle East.

**Methods:**

Clinicians and trainees at Hamad Medical Corporation (HMC) matriculate from many countries in the Middle East and North Africa (MENA) and Asia. Because trainees in Graduate Medical Education (GME) come to HMC from a variety of geographic backgrounds, it is assumed that they also have a variety of experiences and aptitudes in EBM. To assess trainees EBM attitudes and knowledge in the internal medicine department at HMC in Doha, Qatar, the authors surveyed residents and fellows using a two-part survey. The first part was adapted from the evidence-based practice inventory by Kaper to assess trainees’ attitudes and perceptions of EBM. Trainees were also asked to complete the Assessing Competency in Evidence Based Medicine (ACE) tool to evaluate their aptitude in different elements of EBM. The results from the two parts were analyzed.

**Results:**

The average score on the ACE tool among the participants was 8.9 (±1.6). Most participants rated themselves as beginners or intermediate in their EBM capabilities. Higher ACE scores were observed from participants with educational background from South Asia, and among those with more favorable attitudes towards EBM. There was no clear pattern that early incorporation of EBM into practice will result in better ACE score. Participants also reported reasonable abilities in EBM tasks and a favorable work atmosphere for EBM implementation. Lack of knowledge, resources, and time were the most reported barriers to utilizing EBM.

**Conclusions:**

While it is clear that participants are enthusiastic about EBM and see it as a useful method for clinical decision making, their aptitude in EBM is not optimal and there are gaps and barriers for them to practice.

**Supplementary Information:**

The online version contains supplementary material available at 10.1186/s12909-020-02341-9.

## Background

Evidence-Based Medicine (EBM) is described as the “integration of the best research evidence with our clinical expertise and our patient’s unique values and circumstances [1].” EBM is an essential component in the clinical decision-making process and continuing education for clinicians [2]. The core competencies of the Accreditation Council for Graduate Medical Education (ACGME-I); adopted by many institutions in the Qatar and the Middle East region to improve educational outcomes and to matriculate residents to fellowship programs in the United States, require training in EBM skills within its Practice-Based Learning and Improvement competency. Specifically, these requirements state that, “Residents must demonstrate the ability to investigate and evaluate their care of patients, to appraise and assimilate scientific evidence, and to continuously improve patient care based on constant self-evaluation and lifelong learning” [3]. Despite EBM’s accepted position in clinical practice, many graduate medical education programs struggle to find methods for instructing trainees and incorporating EBM in the daily routines of learners. Many residency programs utilize journal clubs or workshop formats, but there is not sufficient data to suggest that any one method is superior [4–8].

While EBM is generally established as a component of clinical education in the United States, Canada and Europe, it is more novel to many clinicians in the Middle East and North Africa (MENA) region. Research on EBM practice and utilization in the MENA region is limited [9–12]. Previous studies focusing on EBM in the region have noted that many clinicians have misconceptions about the fundamentals and applications of EBM. Mortada conducted a study in Egypt where many clinicians were found to be lacking in EBM proficiency, despite asserting to be utilizing EBM. Many clinicians lacked knowledge and aptitude in fundamental EBM concepts [13]. Other studies in Kuwait by Buabbas et al., and in Saudi Arabia by Baig et al., found that while clinicians have a very positive attitude towards EBM, they have low proficiency to apply elements of EBM to patient care [14, 15]. Al Wahaibi conducted a study in Oman where many clinicians indicated they felt that many barriers to practicing EBM existed, such as access to evidence resources or time constraints that prevent them from fully applying EBM in clinical practice [16].

The authors are unaware of any studies that have sought to evaluate EBM capabilities of medical trainees in the State of Qatar.

The objectives of this study were to examine trainees’ self-reported background knowledge, attitudes, use and training in EBM, to test their aptitude for EBM using a validated tool and to look for associations between background variables and the aptitude. The results of this study will ultimately help assess the potential gaps in EBM training and potential areas for future improvements.

## Methods

### Study setting

This study was conducted among internal Medicine (IM) trainees at Hamad Medical Corporation (HMC). HMC is the main healthcare provider in the state of Qatar, comprising of 12 hospitals that provide all levels of care. HMC hospitals serve as the main teaching hospitals to undergraduate training and the only institution in Qatar that offers graduate medical education [16]. HMC is the main affiliate to Weill Cornell Medicine in Qatar (WCM-Q) where students receive their clinical training and clinical faculty have clinical practices. Many HMC consultants have affiliate faculty appointment at WCM-Q. HMC received ACGME-International (ACGME-I) accreditation in 2011. The internal Medicine residency program received accreditation in 2013, one of the first programs in the Middle East. ACGME-I accreditation is based on the standards for ACGME accreditation of teaching hospitals and medical centers in the United States, requiring base standards for trainee programs, including medical knowledge, medical skills, communication, practice-based learning, systems-based learning and professionalism [17]. Other residency programs in the Gulf Region have adopted the CanMEDS framework which was developed by the Royal College in Canada [18]. The residency training requires an internship, in addition to the standard requirements (standardized exam and interviews) to enroll applicants in its four-year training. HMC’s IM residency program attracts trainees from the MENA region with diverse educational and cultural experiences.

This cross-sectional study utilized two survey instruments. The first instrument was used to collect participants’ demographic, educational background, utilization, and attitudes regarding EBM. This instrument was adapted from The *evidence-based practice inventory* developed by Kaper, et al., and adjusted to fit the first aim of this study [19]. Affective elements from Kaper, et al’s survey were incorporated into this study with consideration for keeping the survey brief. Additional survey questions were added to assist in understanding how trainees’ access and aptitude with information resources affected EBM, since this was perceived to be potentially a significant factor in this study. After the authors reached consensus, the survey was piloted on five clinicians who were asked to give feedback on the content and wording of the survey. Minor adjustments were then made.

To assess EBM aptitude, the authors reviewed several tools, ultimately selecting the Assessing Competency in Evidence Based Medicine (ACE) Tool developed by Illic et al., for its established validity and ease of administration [20]. The ACE tool consist of a sample scenario, search, hypothetical article, and 15 binary (yes/no) questions. Each question is assessed one point (max 15pts) to produce a final quantitative score for assessment of EBM knowledge and aptitude.

Execution of this cross-sectional study took place between July 2018 and April 2019 at HMC in Doha, Qatar. The study population was comprised of interns, residents, and fellows within the Internal Medicine department. A pool of 185 potential participants were available for inclusion in this study. This pool is representative of the total available IM trainees from the internal medicine department during the duration of data collection of the study. Participation in this study was requested at education activities, via email, and through snowball methods. Participants were asked to complete the survey (26 total questions) addressing their educational background, use, and attitudes regarding EBM. Self-reported comfort levels for each EBM component were obtained using a five-point Likert scale, with 1 representing “least capable” and 5 representing “most capable.” Participants were asked to self-rate their overall EBM ability as beginner, intermediate, or advanced. In addition, self-reported perceptions of institutional attitudes and barriers to implementing EBM were obtained. These data points were also derived from Likert scales, with 1 representing a negative view and 5 the most positive. Individual attitudes to EBM implementation to clinical care were also asked with answers on Likert scales with 1 indicating a negative attitude and 5 being highest positive attitude. Participants’ preferences for information resources for searching for clinical evidence as an EBM process was also gathered. Participants on those final questions could indicate multiple answers. Following the survey on attitudes and self-ratings, participants completed the ACE Tool to gauge their EBM aptitude. Participants completed the Survey and ACE Tool either through paper-based or online submissions. Qualtrics survey tool was used to collect data. Online submissions were entered directly into the survey tool, while paper-based submissions were subsequently entered into the online survey tool by a member of the research team.

### Ethical consideration

Prior to survey execution, this research, including protocol, survey and recruitment materials, was approved by the institutional review boards of both Weill Cornell Medicine – Qatar and Hamad Medical Corporation. Consent was obtained, either in writing or electronically, from each study participant before initiating the survey and ACE tool.

### Statistical analysis

Demographics and work-related variables are summarized using frequency distributions. Similar summary statistics are used for variables related to EBM, including education, practice, attitudes and, self-perceived ability.

For each participant, the ACE score is computed as the sum of the number of correct answers out of the 15 ACE questions. Some participants (*n* = 10) did not answer the ACE tool in its entirety, with 6 participants only completing 14 of 15 questions and 4 participants completing 13 of 15 questions. For those participants, the unanswered questions were considered as wrong answers.

To assess the potential association between the ACE score and other variables in the study such as demographic variables, self-rated abilities in EBM and time incorporating EBM into practice, the mean ACE score and standard deviation was computed for each category within those variables.

## Results

A total of 94 trainees submitted a response to the study instrument. However, 14 respondents did not go beyond answering the demographic questions and hence were excluded from the analysis since they contributed no potential data to the results. The final sample size included 80 trainees (response rate = 43.2%).

The majority of the participants were age 20–29 (83.8%) and male (65.8%). The majority obtained their medical education in the Middle East Region (60.6%). Interns accounted for 11.3% of participants and fellows accounted for 8.8% (Table 1).

**Table 1:** Participants’ demographics and their ACE Score

			ACE Score	
	N	%	Mean	SD
Age				
20–29	67	83.8%	8.9	1.6
30–39	13	16.3%	9.2	1.4
Gender				
Male	52	65.8%	8.9	1.5
Female	27	34.2%	8.9	1.7
Level of experience				
Intern	9	11.3%	7.9	1.6
PGY1	35	43.8%	8.7	1.3
PGY2	11	13.8%	10.0	1.3
PGY3	10	12.5%	8.6	2.0
PGY4	8	10.0%	10.0	1.8
Fellow	7	8.8%	8.7	1.6
Region from where the Medical Education was received				
Middle East (including Sudan)	48	60.6%	8.7	1.6
North Africa	12	15.0%	8.2	1.3
South Asia	18	22.5%	9.9	1.4
Other^a^	2	2.5%	8.5	0.7

^a^1 Eastern Europe and 1 unknown

Overall, participants’ scores on the ACE tool ranged from 5.0 to 12.0 (out of 15) with an average of 8.9 ± 1.6 and a median of 9.0. This means that participants were able to correctly answer, on average, only 59.3% of the ACE questions. There were no apparent differences in the ACE scores between the two age groups or between genders. Although Interns had the lowest average ACE score among all other groups, there was no major pattern indicating, for example, that an increase in work experience might have a positive impact on the ACE score. For example, average ACE scores for PGY 2 (10.0 ± 1.3) and PGY 4 (10.0 ± 1.8) were slightly higher than that of the Fellows group (8.7 ± 1.6). Those with a medical educational background from South Asia scored the higher on the ACE tool than those graduating from other regions. The biggest difference was between South Asian graduates and those graduating from North Africa, with a difference of 1.7 points out of 15 questions (see Table 1).

Table 2 includes information about participants’ EBM education and its incorporation into practice. Most of the participants learned about EBM in their undergraduate medical education (63.7%) utilizing mainly a mix of face to face and online learning modalities or only face to face (86.3%). The majority started incorporating EBM in their clinical decision-making processes during residency (61.3%). Those who learned about EBM during undergraduate medical education or during residency scored higher; by about 1.75 points, on the ACE tool as compared to those who reported that they haven’t learned about EBM. There was no clear pattern that early incorporation of EBM into practice will result in better ACE score. For example, those who reported not incorporating EBM into their practice had a higher average ACE score; by 1.1 points, compared with those who incorporated it after residency. There was no clear indication that the type of instructional setting has a major impact on the ACE score (see Table 2).

**Table 2:** EBM Educational, background and incorporation into practice and ACE score results

	N	%	ACE Score	
			Mean	SD
At what stage of your medical career did you first learn about EBM?				
During undergraduate medical education	51	63.7%	8.9	1.4
During residency	26	32.5%	9.2	1.9
I have not learned about EBM	3	3.8%	7.3	1.5
In what instructional setting did you learn EBM? - Selected Choice				
Face to face (traditional classroom setting)	30	37.5%	8.6	1.5
Online (eLearning)	4	5.0%	9.0	0.0
Mix of online and face to face	39	48.8%	9.3	1.7
Self-study	6	7.5%	8.2	1.5
Other (not specified)	1	1.3%	7.0	–
When did you begin incorporating EBM within your clinical decision-making process?				
Since undergraduate medical education	16	20.0%	9.4	1.3
During residency	49	61.3%	9.0	1.6
After residency (fellowship & clinical practice)	9	11.3%	7.6	1.9
I have not incorporated EBM within my practice	6	7.5%	8.7	1.2

The self-reported comfort levels for each EBM component and for overall EBM ability are presented in Table 3. For most categories, participants rated themselves as either 3 or 4 on a scale of 1 to 5, with 1 indicating least capable and 5 indicating most capable. In all those components, with the exception of applying EBM to a clinical decision, the percentage of participants who indicated that they are most capable did not exceed 12.5% or 1 in 8. Conversely, the vast majority of the participants rated themselves as beginner or intermediate (89.9%) on their overall EBM abilities (see Table 3).

**Table 3:** Self-reported Comfort levels with EBM components, self-rated overall EBM abilities and ACE score results

outcome is the average score on Ace for each participant	N	%	ACE Score	
			Mean	SD
Applying EBM principles in my clinical decisions				
Least capable	0	0.0%		
2	3	3.8%	7.7	1.5
3	27	33.8%	8.6	1.9
4	36	45.0%	9.1	1.3
Most capable	14	17.5%	9.4	1.4
Translating my information needs into relevant and feasible clinical questions				
Least capable	0	0.0%		
2	6	7.5%	8.7	2.0
3	23	28.7%	8.8	.7
4	42	52.5%	9.1	1.5
Most capable	9	11.3%	8.3	1.7
Searching for research evidence in literature				
Least capable	1	1.3%	7.0	–
2	4	5.0%	8.8	1.9
3	31	38.8%	8.7	1.6
4	34	42.5%	9.2	1.7
Most capable	10	12.5%	8.7	0.9
Critically appraising research evidence from literature				
Least capable	4	5.0%	8.0	2.2
2	17	21.3%	8.5	1.6
3	26	32.5%	8.9	1.7
4	29	36.3%	9.2	1.5
Most capable	4	5.0%	9.8	1.0
Translating research evidence to the care of my individual patients				
Least capable	2	2.5%	7.5	3.5
2	9	11.3%	8.0	1.7
3	21	26.3%	8.5	1.7
4	41	51.2%	9.3	1.4
Most capable	7	8.8%	9.7	0.8
Of regularly keeping up with latest research evidence from literature				
Least capable	4	5.0%	6.0	2.0
2	12	15.0%	8.7	1.3
3	28	35.0%	9.0	1.5
4	29	36.3%	9.4	1.4
Most capable	7	8.8%	8.4	1.3
Rate your overall abilities in EBM				
Beginner	30	38.0%	8.5	1.7
Intermediate	41	51.9%	9.1	1.6
Advanced	8	10.1%	9.6	1.2

There was a trend of increased average ACE scores with increased self-rating on applying EBM principals in clinical decision making, translating research evidence to the care of patients, critical appraisal of research evidence from literature, and overall ability in EBM. On those questions, difference between those who reported least capable (or beginner) and those who reported most capable (or advanced) ranged between 1.1 to 2.2 points. For the other questions, the increasing trend was observed except for a decrease in the score for the group who self-rated themselves as most capable (Table 3).

Participants were asked to rate their perceptions of institutional attitudes and barriers to implementing EBM (Table 4), the majority of participants gave the highest two possible scores; on a Likert scale from 1 to 5, for those questions indicating a general level of encouragement to apply EBM (59.2%), giving attention to EBM application in clinical decision making (72.6%), a strong level of support from supervisors to apply EBM within clinical decisions (77.2%) and a general atmosphere of frequent discussion of research evidence (57.6%).

**Table 4:** Attitudes and Barriers to EBM practice

	N	%
My colleagues [...] me to apply EBM principles in my clinical decisions.		
Discourage	0	0.0
2	1	1.9
3	21	38.9
4	20	37.0
Encourage	12	22.2
In my department, we pay [...] attention to applying EBM principles in our clinical decisions		
No	0	0.0
2	2	2.5
3	20	25.0
4	37	46.3
A lot of	21	26.3
Supervisors in my department [...] me to apply EBM principles in my clinical decisions		
Hinder	0	0.0
2	3	3.8
3	15	19.0
4	30	38.0
Support	31	39.2
My colleagues and I [...] discuss research evidence from literature.		
Rarely	0	0.0
2	9	11.3
3	25	31.3
4	35	43.8
Frequently	11	13.8

When questioned about participants’ attitudes to EBM implementation to clinical care (Table 5), almost all participants gave the two highest scores for EBM usefulness to improving patient outcomes (96.3%), for improving their clinical decisions (93.7%) for feeling that there is a synergy between EBM and their own clinical experience (87.3%). Finally, most participants identified their view of EBM’s most significant limitation, with 51.2% reporting not knowing how to practice EBM, 36.3% citing lack of available resources, 28.7% identifying time limitations, 16.3% reporting lack of support of colleagues and 3.8% reporting lack of support from administration. Except for a single instance with one participant, those who reported the highest two levels of positive attitudes towards EBM had on average higher score on the ACE tools. Again, the maximum difference between any of those two later groups and the other groups didn’t exceed 2.1 on the ACE score.

**Table 5:** Attitude toward EBM in clinical use and relation to ACE score

	N	%	Total ACE Score	
			Mean	SD
I feel that Evidence Based Medicine (EBM) is [useless/useful] to improve my patients’ outcomes.				
Useless	0	0.0%	–	–
2	0	0.0%	–	–
3	3	3.8%	7.0	1.7
4	18	22.5%	9.1	1.5
Useful	59	73.8%	9.0	1.6
I feel that EBM [worsens/improves] the quality of my clinical decisions.				
Worsens	0	0.0%		
2	1	1.3%	9.0	–
3	4	5.1%	7.8	2.1
4	20	25.3%	8.7	1.8
Improves	54	68.4%	9.1	1.5
I feel that EBM [disregards/incorporates] my clinical experience.				
Disregards	0	0.0%		
2	3	3.8%	8.0	1.0
3	7	8.9%	7.3	1.8
4	25	31.6%	9.3	1.6
Incorporates	44	55.7%	9.1	1.5

The top 4 reported resources for searching for clinical evidence as an EBM process were PubMed (82.5%), Google (55%), Google Scholar (40%) and Wikipedia (30%). The most reported reason for selecting the resources of choice was due to ease of use (82.5%) and availability of articles (52.5%) (see Appendix).

## Discussion

The increasing emphasis on ACGME-I standards in the region warrants more attention to the incorporation of evidence in the clinical decision-making process. Although the IM residency training at HMC encourages EBM through implementation of regular journal clubs, the diverse and varied educational backgrounds of trainees makes it essential to assess trainees’ incoming level of EBM knowledge and tailor the EBM instructions to match trainees’ needs. Participants knowledge, as assessed by the ACE tool, showed an average score of 8.9 out of 15 indicating that on average participants correctly answered 59.3% of the questions. There was no clear associations between demographic variables and the ACE score or clear pattern that early incorporation of EBM into practice will result in better ACE score. The ACE score exhibited increasing trends with some of the variables especially the self-rated EBM capabilities and positive attitudes toward EBM but difference didn’t exceed 2.1 (14%) points out of 15.

Participants reported reasonable capabilities of practicing EBM with only 10.1% self-rating themselves as experienced in EBM. Participants also reported a favorable atmosphere in their work environment for EBM implementation. Lack of knowledge, resources and time were the most reported barriers for doing EBM. In some instances, the trend of increasing ACE scores with increasing self-rating EBM capabilities or with more favorable attitudes toward EBM was not complete where we observed a dip in the ACE score for those with highest self-rating and highest favorable attitudes. This might be due to selection bias and the low number of participants in general and particularly in some of the categories defined by the self-rating or attitudes.

In comparison to the study in Australia that validated the ACE Tool, the average ACE tool score of trainees in the Qatari sample was between the means of the participants with novice and intermediate levels of experience, defined by authors as having 2 and 3 years of EBM training respectively. In the study of medical trainees from Australia novice and intermediate trainees scores were (means scores of 8.6 and 9.5 respectively) lower than that of the advanced EBM group in that study (mean = 10.5) [20]. This might be due to the fact that about 40% of the participants in Qatar did not formally encounter EBM until residency and thus they did not have the three to 4 years training in EBM that the advanced group in Australia had.

Although it may be obvious that learning about EBM earlier increases aptitude, there was not a solid trend that early incorporation of EBM within clinical decision-making increases aptitude. This could be due to participants’ diversity in educational backgrounds, not knowing the frequency and details of such incorporations, the potential confounding effect of other variables that the study could not control for due to the small sample size.

This is even more pronounced when comparing the number of participants who incorporated EBM within their clinical decision during undergraduate education (20%), with those whose first instructional contact with EBM was during graduate education (64%). This gap would seem to indicate that a large proportion of participants were exposed to EBM education in a nominal or uncontextualized way. This is congruent with a systematic review that indicated that standalone teaching was not as effective as clinically based teaching in improving residents’ skills, attitudes and behaviors [4]. This is also consistent with the literature, which indicates that, in general, EBM instruction increases knowledge and skills but does not itself impact on physician behavior or clinical practice [21–25].

Most of the participants in this study reported positive to very positive attitudes, both collegially and individually. This is congruent with other studies in the region that showed that clinicians generally have a favorable view of EBM [13, 26, 27]. These studies also cited that despite having positive attitudes towards EBM, this was not necessarily translated into aptitude or knowledge. This was also the case in our study, as participants’ attitudes were very positive to positive, but the average score on the ACE tool was 8.9 out of 15. Additionally, the difference between those with the highest positive attitudes and those with lower positive attitudes was minimal. Besides lacking experience, some of the reasons for this could be the need to guide clinicians about appropriate resources for identifying research evidence and providing them with the protected time to learn. This is evident from participants responses to the questions about EBM’s limitations.

### Strengths and limitations

This study has several limitations. This is a cross- sectional study that was conducted at a finite point in time, as such it lacks the depth that a longitudinal study would afford. In future considerations of this research, it would be helpful to examine not only the EBM aptitude levels of residents at a particular point in time, but how their knowledge changes over the course of their graduate medical education. Additionally, it would be valuable to study what instructional methods work best at increasing residents’ knowledge and application of EBM within the clinical setting. The small sample size of 80 contributed to lack in depth in the analysis, such as assessing the effect of potential confounding variables on the results. Moreover, since participation was voluntary and with a response rate of 43.2%, the results of this study should be interpreted with caution and might not be generalizable to the whole population. Finally, since a lot topics are self-reported rating and self-reported attitudes and behaviors, respondents might have different ways of interpreting them, a limitation that the authors had no control over.

However, this study has several strengths. To our knowledge, this is the first study in the State of Qatar and one of the very few in the Middle Eastern / Arab region to look into the perceptions and attitudes of trainees towards EBM at an ACGME-I program. The use of a validated ACE tool helps in making sure that the measure of the actual ability of participants to practice EBM is accurate. This was evident in several increasing trends observed in ACE scores for some of the self-reported EBM capabilities.

## Conclusion

Although results of such study should be interpreted with cautions due to the limitations described above, this study still offers an interesting insight into perceptions, attitudes and aptitudes among trainees in the State of Qatar. While it is clear that participants are enthusiastic about EBM and see it as a useful method for clinical decision making, their aptitude in EBM is not optimal and there are gaps and barriers for them to practice. Since health care trainees in Qatar come from a diverse cultural and education backgrounds, assessments of EBM abilities and support to improve such capabilities should be in place during their time in Qatar. As such, there should be more emphasis on identifying gaps in individual learner’s knowledge through assessments at initiation and providing time and resources to advance them to a standard level. Graduate medical education institutions can play an important role in identifying the best practices for educating residents about EBM and help in testing such potential interventions.

### Supplementary Information

**Additional file 1.** Study Questionnaire.

## Data Availability

The datasets generated and/or analyzed during the current study are not publicly available due institutional guidelines, but are available from the corresponding author on reasonable request.

## Appendix

**Table 6:** Resources and limitations^a^

Variable	Answers	N	%
Resources used	Pubmed	66	82.5%
	Google	44	55.0%
	Google Scholar	32	40.0%
	Wikipedia	24	30.0%
	Uptodate	22	27.5%
	Medline	19	23.8%
	Medscape	5	6.3%
	Embase	4	5.0%
	Scopus	2	2.5%
	Clinical Guidelines	1	1.3%
	ncbi	1	1.3%
	Wolters Kluwer UTD	1	1.3%
	USMLE forums	1	1.3%
	MICSAP	1	1.3%
	online questions	1	1.3%
	dynamed	1	1.3%
	4mboss	1	1.3%
Why you use specific resources	ease of use	66	82.5%
	availability of articles	42	52.5%
	I don’t know how to use anything else	16	20.0%
	don’t have anything else	13	16.3%
	this is what I use for everything, don’t want to learn something new	3	3.8%
	regularly updated	1	1.3%
	usually find the answers there	1	1.3%

^a^participants could choose multiple answers and that is why percentages will add up to more than 100% per question

## Abbreviations

EBM: Evidence Based Medicine
ACGME: Accreditation Council for Graduate Medical Education
ACGME-I: ACGME International
PGY: Post Graduate Year
HMC: Hamad Medical Corporation
HMH: Hamad Medical Hospital
