# Correction to: Examining aptitude and barriers to evidence-based medicine among trainees at an ACGME-I accredited program

**DOI:** 10.1186/s12909-020-02399-5

**Published:** 2020-11-26

**Authors:** Mai A. Mahmoud, Sa’ad Laws, Antoun Kamel, Dabia Al Mohanadi, Ahmed Al Mohammed, Ziyad R. Mahfoud

**Affiliations:** 1 Weill Cornell Medicine in Qatar, Education City, P.O. Box 24, 144 Doha, Qatar; 2 Hamad Medical Corporation, Doha, Qatar

**Correction to: BMC Med Educ 20, 414 (2020)**

**https://doi.org/10.1186/s12909-020-02341-9**

Following publication of the original article [1], the authors identified an error in the author name of Antoun Kamel.

The incorrect author name is: Antoun Kamal

The correct author name is: Antoun Kamel

The original article has been corrected.
