# Supplementary material for: Examining aptitude and barriers to evidence-based medicine among trainees at an ACGME-I accredited program
Source: BMC Med Educ. 2020 Nov 10;20:414. doi: 10.1186/s12909-020-02341-9 (PMC7654012; doi:10.1186/s12909-020-02341-9)
Supplement: Supplementary file 1 — Additional file 1. Study Questionnaire. [file 12909_2020_2341_MOESM1_ESM.pdf]

Consent Statement

CS.

Examining aptitude and barriers to Evidence Based Medicine (EBM) within a secondary clinical setting.

Informed Consent Statement

This research is being conducted to examine the following:

1. Understand the breadth and depth of utilization of EBM among practicing clinicians within a hospital setting.

2. Identify what education and training clinicians receive in EBM.

3. Identify internal and external barriers that clinicians perceive in practicing EBM?

You are being requested to participate in this survey because you are a clinician practicing at Hamad Medical Corporation involved in the clinical management and treatment of individual patients.

Your participation in this research project is completely voluntary and you are free to withdraw at any time during the study without any penalty. The research team expects your participation to last approximately 1 – 2 hours. The research team estimate that 150 - 200 participants will take part in this study.

If you agree to be in this study, you will be asked to complete two online evaluations. The first will ask you about your experiences, attitudes and perceptions of EBM. The second will begin by asking you to examine a sample case, search and research paper. You will then be asked to answer 15 yes or no questions to assess your aptitude of EBM. Both surveys will

be linked to one-another and must be completed in one setting. Both surveys will also be completely anonymous and no one from the research team, representatives from Weill Cornell Medicine – Qatar, or Hamad Medical Corporation will be advised of the identities of respondents.

You are free to ask members of the research team about your involvement in this research at any time.

This research is being overseen by the Weill Cornell Medicine in Qatar (WCM-Q) Institutional Review Board ("IRB") and the Hamad Medical Corporation (HMC) IRB.

You may talk to the WCM-Q IRB (at +974 4492 8960 or irb@qatar-med.cornell.edu) and the HMC IRB (at +974 4439 8820 or irb@hamad.qa) if:

- Your questions, concerns, or complaints are not being answered by the research team.
- You cannot reach the research team.
- You want to talk to someone besides the research team.
- You have questions about your rights as a research subject.
- You want to get information or provide input about this research.

Consent. I hereby do grant consent to my participation in the above research study

Yes
No

Demographic Questions

Q1. What is your age?

20-29
30-39
40-49
50-59
60+

Q2. What is your gender?

Male
Female

Q3. Describe your clinical role.

Intern

Resident 1

Resident 2

Resident 3

Resident 4

Fellow

Attending / Consultant

Q4. In what location did you receive your medical education?

Middle East

North Africa

South Asia (Pakistan/India)

North America

[ ] Other

Attitude

Q5. I feel that Evidence Based Medicine (EBM) is [useless/useful] to improve my patients' outcomes. (Please select your level from range)

Useless	2	3	4	Useful
○	○	○	○	○

Q6. I feel that EBM [worsens/improves] the quality of my clinical decisions. (Please select your level from range)

Worsens	2	3	4	Improves
○	○	○	○	○

Q7. I feel that EBM [disregards/incorporates] my clinical experience. (Please select your level from range)

Disregards	2	3	4	Incorporates
○	○	○	○	○

Education

Q8. At what stage of your medical career did you first learn about EBM?

During undergraduate medical education

During residency

After I became a consultant

During fellowship

I have not learned about EBM

Q9. In what instructional setting did you learn EBM?

Face to face (traditional classroom setting)

Online (eLearning)

Mix of online and face to face

Self study

[ ] Other

Q10. When did you begin incorporating EBM within your clinical decision making process?

Since undergraduate medical education

Since residency

After beginning clinical practice

Since fellowship

I have not incorporated EBM within my practice

Q10B. How long have you practiced as a consultant

0-5 years

5-10 years

10 + years

I am not a cusultant

Perception of Abilities

Q11. How would you rate your overall technical ability? (i.e. ability to effectively use computers, applications, mobile devices, etc.)

No technical competency	2	3	4	Extremely competent
○	○	○	○	○

Q12. Are you aware of any resources available to you when you need assistance with a technical problem?

Definitely yes

Probably yes

Probably not

Definitely not

Q13. Rate how comfortable you are in the following areas.

	Least capable	2	3	4	Most capable
Applying EBM principles in my clinical decisions	○	○	○	○	○
Translating my information needs into relevant and feasible clinical questions	○	○	○	○	○
Searching for research evidence in literature	○	○	○	○	○
Critically appraising research evidence from literature	○	○	○	○	○
Translating research evidence to the care of my individual patients	○	○	○	○	○
Of regularly keeping up with latest research evidence from literature	○	○	○	○	○

Q14. Rate your overall abilities in EBM.

Beginner	Intermediate	Advanced
○	○	○

EBM Process

Q15. What resources do you use to search for clinical evidence? (Check all that apply)

PubMed

Embase

Medline

Scopus

Google

Google Scholar

Wikipedia

Other

Q16. Thinking about the previous question, what is the reason that you use this resource(s)? (Check all that apply)

Its easy to use

I like the articles available in this resource

I don't have anything else available to use

I don't know how to use anything else

This is what I use for everything (I don't want to learn/use anything else)

Other

Q17. Do you write out the steps of the EBM process, such as your PICO?

I write out everything

I write down some parts, if needed

I do everything in my head

I don't do any of these steps

Other

Q18. Rate what degree of importance each of the following has when formulating clinical decisions for a patient.

	Not important	2	3	4	Very important
Clinical guidelines	○	○	○	○	○
Your patient's values and expectations	○	○	○	○	○
Your clinical expertise	○	○	○	○	○
Current research on the condition	○	○	○	○	○

Barriers/Facilitators

Q19. What do you believe are EBM's most significant limitations?

Time limitations

Available resources

Not knowing how to practice EBM

Not enough support from colleagues

Not enough support from administration

Other

Q20. Where do you see most of your patients in a typical week?

Inpatient

Outpatient

Other

Q21. How many individual patients do you see in a typical week?

0

1-4

5-10

11 +

Q22. Of these patients, how many typically have a condition, question or problem that requires you to search clinical literature for answers?

0

1-4

5-10

11 +

Q23. My colleagues [...] me to apply EBM principles in my clinical decisions.

Discourage	2	3	4	Encourage
◯	◯	◯	◯	◯

Q24. In my department, we pay [...] attention to applying EBM principles in our clinical decisions.

No	2	3	4	A lot of
◯	◯	◯	◯	◯

Q25. Supervisors in my department [...] me to apply EBM principles in my clinical decisions.

Hinder	2	3	4	Support
◯	◯	◯	◯	◯

Q26. My colleagues and I [...] discuss research evidence from literature.

	Rarely	2	3	4	Frequently
	◯	◯	◯	◯	◯

ACE Tool of Competency in Evidence Based Medicine

.

Directions: Read through the following information on patient scenario, clinical question, search strategy and article extract before answering the questions that follow.

.

Patient scenario
"Jane is a 42 year-old female Caucasian, who lives with her partner in metropolitan Melbourne, Australia. Jane is a lawyer, who quit smoking three years ago, after being a 'pack-a-day' smoker since her early 20s. Since her late 30s, Jane has received treatment for hypertension. Her medical history is otherwise unremarkable. At her most recent visit to her family doctor, Jane mentions that she has seen reports on the television about a new study investigating the preventive effects of aspirin. She has heard that aspirin may be beneficial in protecting against cardiovascular disease. Jane wonders whether she should be taking aspirin, given her history with hypertension, but wonders whether also being a diabetic might negate any benefit."

.

Clinical Question
"Is aspirin effective in reducing the risk of cardiovascular disease?"

.

The following is a search used in PubMed
Search Details: (Aspirin OR "Aspirin"[Mesh]) AND (cardiovascular OR "Cardiovascular Diseases"[Mesh]) AND (hypertension OR "Hypertension"[Mesh]) AND (diabetes OR "Diabetes mellitus"[Mesh])
Filters Used: Randomized Controlled Trial; Female; Adult: 19+ years

.

Article extract (hypothetical article)
A randomized controlled trial of aspirin for the prevention of cardiovascular disease
Background

Aspirin is effective in the treatment of acute myocardial infarction and prevention of cardiovascular disease in men and women. Previous studies on the use of aspirin in primary prevention of cardiovascular disease have demonstrated a positive effect in men, yet the benefit in women remains uncertain. The aim of this study was to assess the effect of aspirin in the prevention of cardiovascular disease in women.

Methods

The study design was a randomised, double-blinded, placebo-controlled, trial of low-dose aspirin in the prevention of cardiovascular disease in women. The design of the study has previously been described in detail. In brief, between January 2002 and January 2012, letters of invitation were mailed to 500,000 women in the greater city of Melbourne, Victoria, Australia. A total of 63,250 volunteered to enrol in the study. Women were eligible if they were 40 years of age or older; had no history of coronary heart disease, cerebrovascular disease, no previous side-effects to taking aspirin and were not currently taking aspirin or any non-steroidal anti-inflammatory drug (NSAID) medication. A total of 31,150 women met the inclusion criteria of which 15,100 were randomised (through the generation of a computer generated scheme) to receive aspirin and 15,102 were randomised to receive the placebo. Written informed consent was obtained from all participants prior to commencement in the study. The trial was approved by the ethics board at the governing hospital and university institution. Participants in both groups were required to present every 6 months at the study site centre for assessment and to receive their medication. Medication was provided by the site pharmacy, which allocated identical appearing aspirin and placebo tablet in blister packs to the study's participants independent to the study's investigators. All participants were followed for myocardial infarction, stroke or death from cardiovascular causes. Medical records were obtained for all women in whom a cardiovascular event was recorded. These records were reviewed by an end-point committee, consisting of study investigators blinded to the treatment. The primary end point was cardiovascular events – a combination of myocardial infarction, stroke or death from cardiovascular causes. Only confirmed end-points of cardiovascular events were included in this study. Cox proportional hazard models were used to calculate hazard ratios and 95% confidence intervals for the comparison of event rates in the aspirin and placebo groups after adjustment for age.

Results

Both aspirin and placebo groups were similar with respect to baseline characteristics (Table 1). The average duration of follow-up from randomisation to the end of the trial was 4.2 years (range, 2.3 to 5.0 years). Throughout the duration of the trial, drop-outs occurred. Data presented is based on participants that completed the trial during the study period. A total of 422 women in the aspirin group and 478 women in the placebo group had a cardiovascular event (Hazard Ratio, 0.83; 95% confidence interval, 0.77 to 1.01). There was no evidence that any of the cardiovascular risk factors considered, except smoking status and hyperlipidemia, modified the effect of aspirin on the primary end-point.

Discussion

In this large study, involving 63,250 women, a 100 mg daily dose of prophylactic aspirin is associated with a reduced risk of major cardiovascular events. No significant evidence was

found that age, hypertension, diabetes or BMI modified the effect of aspirin. Middle aged women who adhere to a daily low dose of aspirin can significantly reduce the risk of cardiovascular disease. The rate of benefit is large, with a cardiovascular event prevented for every 269 women treated with aspirin.

**Table 1. Baseline characteristics**			
	**Aspirin (N=15,100)**	**Placebo (N=15,102)**	**Total (N=30,202)**
**Age (years)**			
**(mean±SD)**	55.3±8.0	54.9±8.0	55.1±8.0
40-50 (%)	50.2	50.1	50.1
51-60 (%)	42.9	43.0	43.0
>61 (%)	6.9	6.9	6.9
**Smoking status**			
**Current (%) +**	15.0	14.7	14.9
**Past/never (%)**	85.0	85.3	85.1
**Body mass index (kgm-2)**			
**(mean±SD)**	25.1±4.3	25.3±4.3	25.2±4.3
**<25.0 (%)**	48.8	48.8	48.8
**25.1-29.9 (%)**	32.1	32.2	32.2
**>30.0 (%)**	19.1	19.0	19.0
**Hypertension**			
**Yes (%)**	25.0	24.9	25.0
No (%)	75.0	75.1	75.0
**Diabetes**			
**Yes (%)**	2.3%	2.2%	2.2%
**No (%)**	97.7%	97.8%	97.8%
**Hyperlipidemia**			
**Yes (%)**	27.3	27.2	27.2
No (%)	72.7	72.8	72.8

Mean differences tested using independent t-test; proportional differences tested using the chi square test. $^{+}$significantly different at $p<0.05$

**Table 2. Hazard ratios of cardiovascular events, related to baseline characteristics**				
	**Total number**	**Aspirin**	**Placebo**	**HR (95% CI)**
**Age (years)**				
40-50	15131	122	142	0.86 (0.67-1.09)
51-60	12987	148	166	0.89 (0.71-1.13)
>61	2084	152	170	0.90 (0.74-1.11)

**Smoking status**				
Current	4500	159	140	1.12 (1.00-1.40)
Past/never	25702	263	338	0.78 (0.66-0.92)
**Body mass index (kgm-2)**				
**<25.0**	14738	181	208	0.87 (0.71-1.06)
**25.1-29.9**	9725	150	169	0.97 (0.71-1.11)
**>30.0**	5739	91	101	0.90 (0.68-1.20)
**Hypertension**				
Yes	5051	221	250	0.89 (0.75-1.06)
No	25151	201	228	0.87 (0.73-1.06)
**Diabetes**				
Yes	664	58	62	0.94 (0.68-1.31)
No	29538	364	416	0.87 (0.76-1.01)
**Hyperlipidemia**				
Yes	8214	196	168	1.15 (1.04-1.48)
No	21988	226	310	0.73 (0.62-0.87)

ACE-1. Are all PICO elements described in the patient scenario?

YES

NO

ACE-2. Does the question constructed post-scenario provide a focused, clinical question?

YES

NO

ACE-3.
Will the search strategy (to be used in PubMed) retrieve relevant studies relating to the question?

YES

NO

ACE-4.
Does the search strategy utilise appropriate MeSH/keywords and Boolean operators correctly and effectively?

YES

NO

ACE-5.
Was there sufficient information to determine the representativeness of the study participants?

YES

NO

ACE-6.
Was the method of participant allocation to intervention/exposure and comparison adequate?

YES

NO

ACE-7.
Was any form of adjustment required?

YES

NO

ACE-8.
Were all participants blinded to the treatment/exposure?

YES

NO

ACE-9.
Were all investigators blinded to the treatment/exposure?

YES

NO

ACE-10.
Were all outcome assessors blinded to the treatment/exposure?

YES

NO

ACE-11.
Were all patients analyzed in the groups to which they were randomized?

YES

NO

ACE-12.
Does the patient in the scenario share similar characteristics/circumstances to participants in the study?

YES

NO

ACE-13.
Is the treatment/therapy feasible in the clinical setting of the scenario?

YES

NO

ACE-14.
Were all clinically important outcomes considered?

YES

NO

ACE-15.
Do the likely benefits of the treatment/therapy outweigh any potential harms and costs?

YES

NO

Block 9

Powered by Qualtrics
